# “Lights and Shadows”: An Interpretative Phenomenological Analysis of the Lived Experience of Being Diagnosed With Breast Cancer During Pregnancy

**DOI:** 10.3389/fpsyg.2021.620353

**Published:** 2021-04-01

**Authors:** Federica Facchin, Giovanna Scarfone, Giancarlo Tamanza, Silvia Ravani, Federica Francini, Fedro Alessandro Peccatori, Eugenia Di Loreto, Andrea Dell’Acqua, Emanuela Saita

**Affiliations:** ^1^Department of Psychology, Catholic University of the Sacred Heart, Milan, Italy; ^2^Gynecology Unit, Fondazione IRCCS Ca’ Granda, Ospedale Maggiore Policlinico, Milan, Italy; ^3^Faculty of Psychology, Catholic University of the Sacred Heart, Milan, Italy; ^4^Fertility and Procreation Unit, Division of Gynecologic Oncology, European Institute of Oncology IRCCS, Milan, Italy

**Keywords:** qualitative research, Interpretative Phenomenological Analysis, cancer diagnosis, breast cancer during pregnancy, lived experience

## Abstract

Cancer diagnosed during pregnancy is a rare event. The most common type of malignancy diagnosed in pregnant women is breast cancer, whose incidence is expected to raise in the next future due to delayed childbirth, as well as to the increased occurrence of the disease at young age. Pregnant women diagnosed with breast cancer are exposed to multiple sources of stress, which may lead to poorer obstetric outcomes, such as preterm birth and low birth weight. In addition, pregnancy involves physiological changes in the breasts that may blur the signs of cancer, with delayed diagnosis and poor prognosis. However, the lived experience of these women was investigated in very few studies. Given this scenario, we conducted this qualitative study to describe and understand women’s subjective experience of being diagnosed with breast cancer during pregnancy. The study was conducted following the principles of Interpretative Phenomenological Analysis. Participants were five women with breast cancer diagnosed during pregnancy, purposefully recruited at a public hospital during medical visits and interviewed at treatment initiation. The interview transcripts were analyzed using thematic analysis. The textual analysis led to the identification of three main themes related to: (1) the emotional storm experienced after cancer diagnosis, and the importance of receiving appropriate information and being focused on treatment decisions; (2) physical changes and comparisons with healthy women, associated with feelings of sadness and inadequacy; (3) being positive, feeling free to disclose all kinds of emotions, religion and spirituality as sources of strength. The paradoxical coexistence of pregnancy and cancer represents a stressful experience for women and their loved ones. Adopting a systemic perspective may be important to understand the effects of such a complex condition, also considering its impact on healthcare workers.

## Introduction

Cancer during pregnancy is a rare event that occurs in approximately 1/1,000-2,000 pregnancies (Mitrou et al., 2016; Vandenbroucke et al., 2017). However, a rise in the cases of cancer diagnosis during pregnancy is expected in the next future due to advanced maternal age, increased frequency of obesity, greater awareness of the importance of screening and self-examination, as well as improved diagnostic techniques (Ferrari et al., 2018). The most common types of cancer diagnosed during pregnancy are breast, cervical, hematological, and dermatological cancers, although gastrointestinal, renal, and pulmonary malignancies are also reported (Mitrou et al., 2016; Hepner et al., 2019).

Years ago, pregnant women who received a cancer diagnosis were advised to interrupt the pregnancy, but now treatment (either surgery or chemotherapy) is possible and safe for both the mother and the baby, and cancer during pregnancy does not necessarily have poorer prognosis than non-pregnancy related malignancy (Amant et al., 2015; Ferrari et al., 2018). However, despite these important advances in research and clinical practice, this uncommon condition remains a medical and moral dilemma, which often entails making complex and fast decisions regarding two lives at risk (Cardonick et al., 2015).

Although a huge body of literature provided evidence about the negative psychological impact of cancer, very few studies examined the subjective experience of women with cancer diagnosis during pregnancy. This is surprising if one considers that these women are exposed to multiple sources of stress at the same time. In fact, pregnancy in itself is a major life transition, with remarkable psychological and physical changes that can be emotionally challenging for any woman (Molgora et al., 2020). The co-occurrence of pregnancy and cancer represents a unique condition associated with short- and long-term negative psychological outcomes (Kozu et al., 2020). In a study by Henry et al. (2012), women who were diagnosed with cancer during pregnancy reported greater levels of distress than healthy pregnant women and non-pregnant women with the disease. Most pregnant women with cancer presented clinically significant distress, especially related to intrusive thoughts and anxiety, and long-term symptoms were associated with lack of fertility assistance, pregnancy termination advice, surgery post-pregnancy, cesarean section, insufficient milk to breastfeed, and current recurrence of cancer (Henry et al., 2012).

The dyadic nature of this experience was underlined by Vandenbroucke et al. (2017), who assessed psychological distress and coping strategies in both women and their partners. In this study, women were more inclined to maintain pregnancy than partners, and internalizing coping strategies (such as helplessness/hopelessness and anxious preoccupation) were associated with concerns about the child’s health, about the disease and its treatment, and regarding pregnancy and delivery.

### Breast Cancer During Pregnancy

Breast cancer is the most frequently diagnosed malignancy during pregnancy, since it occurs in 1–4 cases per 10,000 pregnancies, and its prevalence is expected to increase in the next future, at least in developed countries, due to delayed childbearing, and thus advanced maternal age (Alfasi and Ben-Aharon, 2019). Another important risk factor is represented by the greater incidence of breast cancer at young age (Alfasi and Ben-Aharon, 2019; Faccio et al., 2019). It has been estimated that approximately 7–10% of women with breast cancer are aged < 40 years (Rossi et al., 2019). In general, being diagnosed with cancer at young age may lead to adverse short- and long-term psychological outcomes, including poor body image, self-efficacy, and self-esteem (Tonsing and Ow, 2018). However, the specific needs and characteristics of young women with breast cancer—e.g., risk factors, tumor biology, prognosis and clinical outcomes, as well as psychosocial issues related to fertility preservation, sexuality, stress, family and working life—have been explored by a relatively small body of literature (Rossi et al., 2019; Saita and Acquati, 2020).

The available research evidence on breast cancer during pregnancy is even poorer, especially with regards to the psychological implications of this condition and women’s subjective experience. In a broader exploratory qualitative study by Rees and Young (2016), focused on the perspectives of young women with breast cancer, three participants were pregnant at diagnosis. These women experienced disrupted expectations of and plans about their pregnancy, with significant implications for motherhood (e.g., not being able to breastfeed, being concerned about the possible negative consequences of premature birth on the baby) and worries about the impact of cancer treatment on future fertility. At the same time, they felt proud of having been able to come through such a difficult situation.

In another qualitative study focused on the developmental process of becoming mothers in women who experienced breast cancer (Faccio et al., 2020), the gestational breast cancer group (i.e., four women who were diagnosed with breast cancer during pregnancy) reported fear of not being adequate mothers due to the disease and its treatment, as well as fear of not being able to breastfeed their child, and underlined the importance of partner support. This study provided new insights into the themes related to motherhood in this population, but women’s subjective experience of being diagnosed with breast cancer during pregnancy remains underexplored.

It should also be considered that pregnancy is associated with physiological changes in the breasts, and for this reason women may not be able to recognize the symptoms of breast cancer, which may lead to delayed diagnosis and poor prognosis (Alfasi and Ben-Aharon, 2019). In addition, women’s exposure to high levels of stress during pregnancy might be associated with poor obstetric outcomes, such as preterm birth and low birth weight (Witt et al., 2014).

Given this scenario, also characterized by a remarkable paucity of research, we conducted the current qualitative study to provide an in-depth exploration and understanding of women’s subjective experience of being diagnosed with breast cancer during pregnancy, with a specific focus on their emotional reactions, concerns, challenges and resources, and needs after the diagnosis.

## Materials and Methods

### Study Design, Sample, and Data Collection

The findings reported in this article are derived from a larger qualitative research project on women’s subjective experience of cancer diagnosis during pregnancy, which started in July 2019. In this article, we specifically focus on the experience of pregnant women diagnosed with breast cancer. The research was approved by the Institutional Review Board (Comitato Etico Milano Area 2, approval date 3 July 2019, approval number 649_2019bis).

The study was conducted using Interpretative Phenomenological Analysis (IPA), an inductive, idiographic approach characterized by an in-depth analysis of how individuals experience and make sense of major life events (Smith et al., 2009). Purposeful sampling was used to recruit participants who were diagnosed with cancer during pregnancy and were able to understand and speak fluent Italian. Women under the age of 18 were excluded, as well as women with pre-existing diagnosed psychiatric disorders. Eligible participants were recruited by the healthcare providers of the center in which the study was conducted. At the end of the visit, women were extensively informed about all aspects of the research and invited to participate. None of the patients who received our invitation refused to participate in the study and all of them returned signed consent form. One woman was not included in this study because she came from another country and her fluency in Italian was poor.

Relevant information was collected using an in-depth, one to one semi-structured interview. The participants included in this study were interviewed from July 2019 to February 2020 (before the Covid-19 outbreak) in a public hospital. We developed an interview schedule that included open-ended questions, starting with a general, narrative question, such as “Please, could you describe your experience of being diagnosed with cancer during pregnancy?,” aimed at encouraging participants to freely narrate their experience. The topic areas covered by the interview were: (1) experience of the diagnosis, (2) feelings and emotions, (3) effects on relationships, (4) challenges, (5) resources and coping strategies (either on a personal or a relational level). The complete interview schedule has been provided as Supplementary Material. Given the sensitivity of the topic, all interviews were conducted in the form of a conversation with an empathic, dialogic approach by the first author, who is a researcher and a psychotherapist. Women were interviewed individually in a quiet room at the hospital, which ensured privacy. All women were interviewed on the day of chemotherapy, before treatment initiation or during the first days of treatment. The interviews were audiotaped with participants’ consent and subsequently transcribed verbatim. The procedures used to protect confidentiality were carefully explained to all women. These procedures involved assigning a pseudonym to the participant data (therefore, all the names reported in this article were not women’s real names) and removing all identifying details from the transcripts.

From July 2019 to February 2020, all the pregnant patients treated for cancer at the hospital where the study was conducted had breast cancer, which was not surprising, considering that this is the most frequent type of tumor diagnosed during pregnancy (Hepner et al., 2019). The members of the multidisciplinary research team involved in the study agreed that the data collected allowed for a thorough exploration of (and reflection upon) the lived experience of being diagnosed with breast cancer during pregnancy (at least before the pandemic). Thus, we concluded that interviewing additional women with breast cancer would not have significantly changed the findings of this study, also considering the remarkable paucity of research on this topic.

### Data Analysis and Rigor

Textual analysis was performed using an inductive approach, which entails that all emerging themes were derived from the words of our participants, rather than from preconceived theoretical concepts and published research evidence (Smith et al., 2009). Two authors independently performed data analysis without using any software, also considering that the interpretative endeavor made by the researcher through the immersion in the text is essential in the context of IPA (Smith et al., 2009). The multidisciplinary nature of the research team, and the consequent integration of different perspectives and experiences, was also important during the analytic process (Smith et al., 2009). In fact, the findings were constantly shared and discussed with the whole team, which enhanced the researchers’ reflexivity and thus reduced the influence of their preconceptions and biases on the analytic process, with increased rigor (Smith et al., 2009; Larkin, 2018; Fiocco et al., 2020). Field notes were taken during and after each interview as an audit trail and were used to increase the quality of textual analysis.

The analytic process initially entailed line-by-line reading of each interview to provide a preliminary description of relevant topics, with notes recorded directly in the text. These initial notes were then aggregated to identify emerging themes for each participant, and then across participants, with a constant attention to the connection between each interview and the whole corpus of interviews. This circular process led to the identification of a set of superordinate themes and subthemes that in this article are reported as an encompassing narrative, with quotations derived from the interviews to privilege women’s own voices and to show (rather than just tell) their lived experience by providing evocative examples (Adams and van Manen, 2017). English translation of these quotations was performed by the first author and checked for accuracy by all authors.

## Results

Participants were five pregnant women with breast cancer, aged 31–45 years (mean age = 38.2; standard deviation = 5.1). Gestational age at diagnosis ranged from 4 to 26 weeks, and at the time of the study all women were about to start or have recently started chemotherapy. All the participants were Italian and married or cohabitating with their partner. Three participants were expecting their second child, and one of the two primiparous women had undergone IVF.

Textual analysis led to the identification of three dominant themes: (1) overwhelming emotions; (2) sense of difference; and (3) sources of strength. Each dominant theme involved a set of subthemes. All the themes and subthemes (with additional exemplificative quotations) are shown in Table 1.

**TABLE 1:** Themes, subthemes, and exemplificative quotations.

Theme	Subtheme	Exemplificative quotations
1. Overwhelming emotions	Feeling scared and vulnerable	*“I am worried about the consequences of chemotherapy on the baby, but I have been reassured by doctors”* (Joey)
		*“I am worried I might lose my baby, [*…*] this is a high-risk pregnancy. [*…*] I have to get treatment and I am worried that something bad might happen to her”* (Grace)
		*“At first I was scared because I heard that children whose mothers were treated with chemotherapy may have problems up to the age of 10, so I thought*… *what is it going to happen to her?”* (Lucy)
		*“I am worried I might not make it. I don’t want to leave my baby”* (Joey)
		*“My concern is how to explain to him* [the other son] *why I am going to lose my hair, which I have already cut*… *We tried to take it as a game*… *we did this together as a family, to avoid traumatizing him, and now we have started to buy nice hats, such that he can get used to this”* (Grace)
	Feeling shocked and confused	*“I noticed a lump in my breast while I was breastfeeding my baby. I didn’t give much importance to it, I thought it was related to breastfeeding, but then I noticed enlarged lymph nodes in my armpit. I got checked and in a few days I found out it was cancer”* (Joey)
		*“I felt terrible, I didn’t understand anything”* (Faith)
		*“Why me? Why now? Such a misfortune!”* (Lucy)
	Thinking straight and taking action	*“It is tough to carry on a pregnancy under therapy, thinking straight is essential”* (Lucy)
		*“Things have to be done, you can’t just stay in bed, you have to move on”* (Grace)
		*“The sooner I start treatment, the sooner I heal”* (Donna)
		*“There is no need to mince words, you just have to undergo therapy, without too much theory”* (Joey)
2. Sense of difference	Experiencing remarkable physical changes	*“I used to be pretty, but now I have been packing 40 extra pounds and my hair is going to fall out in chunks. He* [husband] *says: Shave it! I try to hold on, but as I touch my head, a tuft of hair falls out”* (Lucy)
	Comparisons with healthy women	*“This was supposed to be a serene time for me as for the other women”* (Faith)
3. Sources of strength	Being positive	*“I just found out that I can be strong*… *I thought I was weaker. So, I want to be positive, because I noticed that after a few tragic days, I was able to react to the situation”* (Donna)
	Feeling supported and listened to	*“My mom is very positive, too. She tells me to stay calm because I will be fine”* (Faith)
		*“They* [girlfriends] *call me every day to know how I feel, to reassure me”* (Faith)
		*“He* [husband] *is always very positive, he cheers me up. He keeps telling me to think positive because things will be alright”* (Grace)
		*“My son gives me strength”* (Grace)
		*“She* [daughter] *is everything to me”* (Lucy)
		*“My doctor called me and said: «It’s been a bombshell, but don’t worry. We have to take care of you and the baby, now. Everything will be fine, you are a lioness»”* (Faith)
		*“If I cry, he* [husband] *cries, and this pushes me down. [*…*] I kept hanging out with friends, going to the gym. I didn’t retreat into myself. I am worried, but I also need distractions”* (Donna)
	Religion and spirituality	*“They are all praying for me and sending me positive messages”* (Faith)
		*“I have always been scared by this disease, in the place where I live cases of cancer occur in almost all families, and I have always thought that maybe, one day, that could be me”* (Faith)
		*“I heard that breast cancer, from a spiritual perspective, may be related to something unresolved in one’s history”* (Lucy)

### Theme 1: Overwhelming Emotions

The first theme related to participants experiencing an emotional storm after the diagnosis. Women spoke about how being diagnosed with cancer during pregnancy involved dealing with a variety of overwhelming, paradoxical emotions. Faith described the experience of being a pregnant woman with cancer as *“a constant alternation of lights and shadows,”* in which the joy for the growth of a new life is overshadowed by the disease. Lucy claimed that *“one forgets being pregnant, because the disease takes over.”* For Joey, who was still breastfeeding her first child when she found out that she was pregnant with her second baby and she also had breast cancer, the diagnosis was “*a punch in the face.*” The complex nature of such an emotional experience was reflected by the three subordinate themes identified: (1) feeling scared and vulnerable; (2) feeling shocked and confused; and (3) thinking straight and taking action.

#### Feeling Scared and Vulnerable

Fear and anxiety for the possible consequences of the disease were reported by all women, and the diagnosis was initially linked to the idea of death by several participants, especially those who were expecting their second child. These women were particularly worried about dying because they did not want to leave their first baby orphan, as clearly expressed by Lucy, who had a 2-year-old daughter, and was diagnosed with breast cancer immediately after discovering she was pregnant with her second child:

*“When you find out that you have cancer, you immediately picture yourself in a coffin, not to mention how you feel if you have a baby*. *Without her, I would probably have reacted differently. But with a baby*… *I am completely focused on her. [*…*] When they tell you this* (the diagnosis), *it may initially sound unbelievable, but you immediately see yourself dead, for sure”* (Lucy).

Donna, who was also pregnant with her second child, explained that, when she thought about the chance of dying from cancer, she felt more worried and sadder for her loved ones than for herself:

*“You have to consider that things* (the treatment) *may not go as you wish, which makes me sad for those who remain, including my baby, rather than for myself. My husband… a baby without her mother*… *I mean, he has to work, his parents live far away from us, it would be complicated. My parents, it hurts to see them suffer. I am not that worried about my own pain. My main concerns are about my close ones”* (Donna).

The potential negative short- and long-term effects of treatment on the health of the unborn child, including miscarriage, were another important source of fear for the participants. Grace, for instance, had troubles conceiving her second child, and for this reason she was particularly worried about the risk of losing the baby, either immediately after the diagnosis (due to pregnancy interruption) or due to the consequences of treatment (see also Table 1):

*“This time, I had troubles becoming pregnant. [*…*] Finally, on the Father’s Day, I discovered that I was pregnant. A week later, I did a breast self-exam and I felt a lump, then I had a needle aspiration biopsy and it was cancer. I feared that I would have to terminate my pregnancy, after so much effort. [*…*] I was convinced that the doctors would have recommended interruption, but instead they told me to try to carry the baby to term”* (Grace).

Faith was pregnant with her first child, and she felt a lump in her breast while spreading stretch mark oil. Her feelings of vulnerability were specifically related to the fact that her tumor was diagnosed when she was pregnant, with a disruptive impact on her hopes and expectations:

*“It shouldn’t have happened now. In a different time of my life, I would have been stronger. It was supposed to be a good time for me”* (Faith).

#### Feeling Shocked and Confused

These feelings were experienced by almost all women immediately before and after the diagnosis, when the situation was still uncertain. In several cases, the signs of the disease were confused with the normal changes related to pregnancy or to breastfeeding (two women got pregnant when they were still breastfeeding their first child; see Table 1). Lack of information was an important source of confusion immediately after the diagnosis, as explained for instance by Donna:

*“That day I was alone. He* (husband) *could not come with me because he had a problem at work, but he optimistically encouraged me saying: «Don’t worry, I am sure it’s nothing». Then I went to the hospital to pick up the results of the exams, and they said they found abnormal cells and started talking about chemotherapy. They tried to reassure me, but I was able to put two and two together [*…*]. I was very confused [*…*]. I wasn’t ready, I didn’t know the incidence of the condition and the prognosis. I fell into despair for a few days”* (Donna).

#### Thinking Straight and Taking Action

For most participants, these feelings of shock and confusion, which led to an initial sense of hopelessness and disorientation, decreased or even disappeared when they received comprehensive information about therapeutic options. At treatment initiation, when the interviews were performed, almost all women said that they felt very focused on the actions they had to take to get out of that painful situation and heal. For example, when Donna claimed there was something strange in her breast, people reassured her that it was due to pregnancy-related changes, but she was not convinced, and she wanted to get it checked. Unfortunately, it was breast cancer:

*“I initially felt desperate, but then we met different professionals, because I wanted to hear other opinions. I talked to three doctors, and they suggested me to meet doctor* (removed), *who is considered the most important Italian expert in cancer during pregnancy. So, I came here, and in 1 week everything was done. From that moment on, I felt much relieved. [*…*] I could not wait to start therapy”* (Donna).

### Theme 2: Sense of Difference

Women’s sense of difference was related to experiencing remarkable physical changes (first subtheme), as well as to comparisons with healthy women (second subtheme) and involved intense psychological sufferance. Overall, this theme highlighted the negative impact of cancer during pregnancy on women’s identity and sense of femininity.

#### Experiencing Remarkable Physical Changes

All participants expressed anguish and sadness related to the consequences of chemotherapy on their body, especially with regard to hair loss, combined with pregnancy-related changes. For example, Lucy knew that chemotherapy was necessary for her, but at the same time she perceived treatment as *“something chemical injected into your body, something that irreparably changes your person.”* Lucy also described the negative effects of chemotherapy on her body, besides losing her hair and getting weight due to pregnancy (as reported in Table 1):

*“Last week I particularly suffered from the side effects of chemotherapy. [*…*] I felt very cold for 15 min and I had to use blankets to warm myself up, I got fever. Then sweat, the fever dropped, and I had to have a shower. [*…*] Then I felt ok for approximately 1 h, and then cold again. It lasted 3 days, I felt exhausted and I was not hungry. Yesterday I felt perfectly fine. You just have to wait it out”* (Lucy).

Faith had always loved and taken good care of her long hair, and it was now difficult for her to accept the idea of losing it. *“It is a major concern for me,”* she cried. Donna too was worried about losing her hair, but she was trying to find alternative solutions, such as choosing the right wig:

*“I don’t want to isolate myself only because I am going to lose my hair. [*…*] I know it might have a strong emotional impact*… *it might cause inhibition*… *but I have already visited several wig stores because I want to buy the perfect wig. I don’t want to look too different; I want it to look natural on me. [*…*] So, I started my search, although people have been telling me to wait. I have already cut my hair a bit shorter. Maybe I am not going to lose it all, but I actually think I will”* (Donna).

#### Comparisons With Healthy Women

Several participants spoke about the fact that they felt different from other healthy pregnant women. In this situation, feeling different meant feeling inadequate as women and mothers, and feeling even sadder for their condition. Grace, for instance, felt stressed and tired, and was no longer able to enjoy playing with her child at the park:

*“I feel more nervous, sometimes I feel more irritable and less patient with him* (child), *I can’t play with him anymore like I used to, I can’t run with him, I feel low in energy. I see the other pregnant women, they are more active than me. [*…*] I am sorry about that, because he* (child) *likes doing somersaults, or jumping on me”* (Grace).

### Theme 3: Sources of Strength

Despite the multiple emotional and physical challenges involved by their condition, women were able to identify important sources of strength that were mostly related to being positive (subtheme 1), feeling supported and listened to (subtheme 2), religion and spirituality (subtheme 3).

#### Being Positive

For almost all participants, having a positive attitude was essential to cope with such a critical situation. For Grace, *“being a smiling person, being able to focus on other things, and thinking positive, despite everything”* were important sources of strength related to her personality. Faith described herself as *“a positive and optimistic person”* and she said she was sure everything would be alright. Donna’s approach to her condition was *“positive, very positive,”* to the point that she was surprised (see Table 1). Lucy on the other hand was more cautious:

*“It’s a huge sacrifice, sometimes you feel down. [*…*] But one can make it. Thus far, I have never felt completely low. I try to find the positive side, but I cry if I want to”* (Lucy).

#### Feeling Supported and Listened to

Being surrounded and feeling supported by positive and optimistic people (including parents, husbands, and friends) was essential for the participants. Among women who were expecting their second baby, the first child represented a special source of joy and energy (see Table 1). During the interviews, women also showed pictures of their babies and talked about their uniqueness, with smiles on their faces. At the same time, women expressed the need to freely disclose all kinds of feelings to their significant others because, as acknowledged by Grace, *“being positive is not always easy and sometimes you just need to let off steam.”* Lucy also claimed:

*“It’s not self-pity. I just want to disclose my emotions, to make you part of this. It’s my kind of letting go”* (Lucy).

For Donna, on the other hand, talking about her negative feelings with her husband was not easy (see Table 1): *“If I cry, he cries, and this pushes me down.”*

Women’s accounts also highlighted the importance of doctors’ capacity to provide support and listen to them. A positive relationship with doctors, characterized by trust and positive communication, was an essential resource for all the participants (see Table 1).

#### Religion and Spirituality

This subtheme explores the role of religion and spirituality in shaping women’s subjective illness experience. Faith talked about the importance of religion in her life and expressed a sense of gratitude toward her community. She said: “*This is God’s will and I am sure everything will be alright.”* At the same time, she had always thought that cancer would have been part of her destiny, because she came from a polluted Italian region with high cancer mortality rates. Lucy thought that her cancer was linked to a past tragic experience and talked about the long-term positive effects of a previous experience with a spiritual guide, which helped her cope with her current situation (see Table 1).

## Discussion

In this study, the experience of being diagnosed with breast cancer during pregnancy as reported by five women was examined in depth using IPA (Smith et al., 2009). The textual analysis of the interview transcripts led to the identification of three dominant themes entitled (1) overwhelming emotions, (2) sense of difference, and (3) sources of strength.

The first theme described the complexity of the experience, characterized by the coexistence of two antithetic conditions: a new life growing inside women’s belly (the light) and a life-threatening disease (the shadow). The paradoxical nature of cancer diagnosis during pregnancy and its negative psychological consequences have been described elsewhere (Henry et al., 2012; Ives et al., 2012; Ferrari et al., 2018). Our findings provide further insight into the nature of this emotional storm. Women tend to perceive cancer diagnosis during pregnancy as a death sentence for themselves and the baby, with an initial reaction of shock and confusion. Pregnancy may enhance their feelings of physical and psychological vulnerability, as also underlined by other authors (Alder and Bitzer, 2008). Receiving clear and comprehensive information from doctors may help women overcome confusion and increase their capacity of “thinking straight” and be focused on the actions they have to take, which is essential immediately after the diagnosis. A positive communication with doctors, with good quality of the information provided, is therefore an important protective factor that may help women cope with cancer during pregnancy, as underlined in previous studies exploring healthcare experiences in this population (Hammarberg et al., 2018). Moreover, our results confirmed that early diagnosis of breast cancer in pregnant women can be difficult, because the manifestations of the disease may be confused with the physiological changes related to pregnancy, which may lead to poorer outcomes (Alfasi and Ben-Aharon, 2019).

Our findings also demonstrated that another source of emotional vulnerability derives from women’s sense of difference (theme 2), related to the significant physical changes caused by the combination of pregnancy and cancer, as well as to comparisons with healthy pregnant women. This situation can result in loss of identity and sense of inadequacy. Body image alterations (especially related to chemotherapy-induced alopecia) and decreased sense of femininity have been acknowledged as important sources of distress in women with breast cancer (Choi et al., 2014; Fingeret et al., 2014). It is also known that body image disturbances in pregnancy are associated with negative health outcomes such as depression, low self-esteem, and impaired maternal-fetal attachment (Nagl et al., 2019). Thus, the risk of distress related to altered body image is doubled among pregnant women with cancer.

Theme 3 showed that having a positive attitude and being surrounded by positive people was helpful for the women in our study. The role of positive thinking (including benefit finding, fighting spirit, and optimism) in helping women cope with cancer has been highlighted in several studies (Carver et al., 2005; Hodges and Winstanley, 2012). However, our participants felt supported and listened to by their significant others when they could freely disclose all their emotions, and even cry if needed (which was not always possible). Women also discussed the importance of religion and spirituality, which underlines the importance of taking care of the spiritual needs of these patients, especially considering that spiritual distress is associated with poorer psychological outcomes in people with cancer (Puchalski et al., 2019).

### Study Limitations

In the context of IPA, the study sample has to be small and homogenous (Smith et al., 2009), and our sample matched these criteria. Moreover, all the participants experienced breast cancer diagnosis before the pandemic. Including pregnant women diagnosed with cancer during the Covid-19 outbreak would have compromised the sample homogeneity due to the psychological impact of the pandemic (the research team discussed a great deal on this issue). However, although it should be considered that breast cancer is the most common form of malignancy among pregnant women (Hepner et al., 2019), the broader experience of cancer diagnosis during pregnancy is more complex, and its essence could not be entirely caught in this small IPA study. In addition, all the participants were interviewed at treatment initiation (or during the initial phases of treatment), which increased homogeneity on the one hand, but on the other hand did not allow for comparisons between women at different stages of therapy (although the focus of this study was women’s experience of being diagnosed with breast cancer, rather than the impact of treatment). Another methodological limitation is represented by the fact that we did not have the chance to discuss our findings with the participants and their partners, or with healthcare providers other than those involved in the research. However, in our study, the multidisciplinary nature of the team represented an importance resource, because the findings were constantly shared and discussed from different perspectives, which enhanced reflexivity.

### Suggestions for Future Research

The knowledge derived from our findings is far from being exhaustive. However, our study may suggest new research avenues to further understand the subjective experience of women diagnosed with cancer during pregnancy. For instance, future studies should examine the characteristics of doctor-patient communication more in depth, since our findings underlined that the quality of patients’ relationship with doctors is essential. Comparisons between women experiencing different types of malignancies should be systematically conducted to examine the associations with specific psychological outcomes, such as for instance maternal-fetal attachment. Moreover, a control group of young patients with cancer diagnosed outside pregnancy could be useful to understand the specific themes related to pregnancy rather than to young age.

Investigating the impact of the diagnosis on family relationships is also important, especially as regards couple relationships. In the context of cancer during pregnancy, partners are even more involved because the disease threatens not only their loved one, but also their child. Examining couple short- and long-term psychosocial adjustment would offer new insights into the understanding of this complex condition. Longitudinal studies are encouraged to investigate the consequences of this stressful experience on the mother-child attachment, as well as on the child’s development.

### Clinical Implications

Our findings may offer suggestions for clinical practice with women diagnosed with breast cancer during pregnancy. First, when communicating the diagnosis, doctors should clarify that treatment is possible and safe for both the mother and the baby, and that in most instances they do not need to terminate the pregnancy. Adopting a participatory stance may help women restore a sense of control by feeling actively engaged in the healthcare process. Our findings suggest that women’s priority immediately after the diagnosis is to know what actions they have to take, which may lead to reduced stress and confusion. In this initial phase, women may feel the need to temporarily put their emotions aside to focus on treatment decisions. In this regard, we believe that adopting a shared decision-making approach, based on a frank patient-doctor dialogue, is essential in the context of cancer during pregnancy (Kozu et al., 2020). Shared decision-making entails providing the patient with comprehensive information about all treatment options (including risks and benefits, and the available research evidence), as well as incorporating her preferences, values and needs into treatment decisions (Katz et al., 2014).

Multidisciplinary treatment programs should be personalized and tailored to women’s physical and emotional needs. Psychological support should be routinely offered to women, and including partners in psychological counseling may be useful. Encouraging a positive attitude may be effective, but it is also important to remind women that feeling sad and crying is normal. Offering them a professional space to freely talk about their experience (which is not always possible with their significant others, who can be very distressed) is essential. Psychological interventions with these patients should also address issues related to women’s identity, including body image and sense of femininity.

## Conclusion

The psychological consequences of cancer diagnosed during pregnancy remain a neglected topic. Adopting a systemic perspective may allow for a better understanding of the effects of this complex condition, also considering its impact on healthcare workers, who are exposed to patients’ physical and psychological pain. The presence of mental health professionals in cancer units is essential, and there is need for research evidence to help healthcare administrators develop appropriate healthcare policies.

## Data Availability Statement

The datasets presented in this article are not readily available because there are privacy and ethical restrictions. Requests to access the datasets should be directed to FFa, federica.facchin@unicatt.it.

## Ethics Statement

The studies involving human participants were reviewed and approved by Comitato Etico Milano Area 2 (approval date 3 July 2019, approval number 649_2019bis). The patients/participants provided their written informed consent to participate in this study.

## Author Contributions

FFa, GS, FP, and ES conceptualized and designed the study, in collaboration with SR and FFr. FFa conducted the interviews and subsequently SR and FFr transcribed the interviews. FFa and ES performed the textual analyses. FFa, SR, and FFr drafted the manuscript. ED and AD assisted in the study conceptualization and the data collection and curation. ED, AD, GS, FP, and GT reviewed the manuscript and provided suggestions related to intellectually important content. All authors read and approved the final version of the manuscript.

## Conflict of Interest

The authors declare that the research was conducted in the absence of any commercial or financial relationships that could be construed as a potential conflict of interest.
