# Supplementary material for: “Lights and Shadows”: An Interpretative Phenomenological Analysis of the Lived Experience of Being Diagnosed With Breast Cancer During Pregnancy
Source: Front Psychol. 2021 Apr 1;12:620353. doi: 10.3389/fpsyg.2021.620353 (PMC8049111; doi:10.3389/fpsyg.2021.620353)
Supplement: Supplementary file 1 [file Data_Sheet_1.doc]

**Interview schedule for women diagnosed with breast cancer during pregnancy**

**Aim:** To examine in depth the subjective experience of being diagnosed with breast cancer during pregnancy

**Topic areas:** (1) experience of the diagnosis, (2) feelings and emotions, (3) effects on intimate relationships, (4) challenges, (5) resources and coping strategies

**Questions**

**(1) Experience of the diagnosis**

--Please, could you describe your experience of being diagnosed with cancer during pregnancy?

**Possible prompts**: What happened? When? How did you discover that you had breast cancer? How was the diagnosis communicated to you? Can you tell me more about that? What do you mean?

**(2) Feelings and emotions**

--What were your thoughts and feelings before / after the diagnosis?

--How did you feel about your pregnancy?

--How do you feel today?

**Possible prompts:** Can you describe your emotional reaction to the diagnosis? Can you tell me more about that? What do you mean?

**(3) Effects on relationships**

Couple relationship:

--Can you describe how you and your partner have been dealing with this situation as a couple?

**Possible prompts:** Do you talk about this with your partner? Do you feel supported by your partner?

Other areas of interest: relationship with the first child (if present); family and social relationships:

--Can you describe how the diagnosis has affected your relationship with your baby / your family / your friends?

**Possible prompts:** Relationships with other people (including doctors)

**(4) Challenges**

--What are your main concerns about being a pregnant woman with cancer?

--What are the main difficulties related to this situation?

**Possible prompts:** How do you feel about that? How do you deal with this?

**(5) Resources and coping strategies**

--What gives you strength in this situation?

--What makes you / what do you do to feel better?

**Possible prompts:** In relation to specific difficult moments narrated by the participants, ask them how they dealt with those difficulties.
